# Supplementary material for: Burnout and associated occupational stresses among Chinese nurses: A cross-sectional study in three hospitals
Source: PLoS One. 2020 Sep 10;15(9):e0238699. doi: 10.1371/journal.pone.0238699 (PMC7482915; doi:10.1371/journal.pone.0238699)
Supplement: S1 Appendix — (PDF) [file pone.0238699.s001.pdf]

中南大学护理学院

行为医学与护理学研究伦理审查委员会

项目名称 **(Project Title)**：Burnout and its influencing factors among nurses :A comparative study between China and Syria
项目负责人 **(Principal Investigator)**：Yasira Kabakleh
伦理评审编号 **(IRB Approval Number)**：2018035
伦理评审日期 **(IRB Approval Date)**：2018.12.30
伦理评审类型 **(IRB Type)**：Convened Review
评审有效期 **(Expiration Date)**：2018.12.30-2019.12.30

此项目是会议审查项目，经中南大学护理学院行为医学与护理学研究伦理审查委员会审议，认为此项目是最小风险项目，符合伦理审查通过的要求，在上述有效期内伦理评审有效。(The above-referenced protocol was approved convened review by the IRB. The project was found to be of minimal risk and to meet the approval requirements under University IRB policy.)

请在项目执行过程中严格遵守医学伦理道德原则，按照研究计划和申请报告进行，保障受试者的权益，并及时向本伦理委员会报告项目执行过程中发生的意外事件和处理情况。

中南大学护理学院行为医学与护理学研究伦理审查委员会
IRB of behavioral and nursing research in School of Nursing of CSU

中南大学护理学院 行为医学与护理学 伦理审查委员会
