# Supplementary material for: Burnout and associated occupational stresses among Chinese nurses: A cross-sectional study in three hospitals
Source: PLoS One. 2020 Sep 10;15(9):e0238699. doi: 10.1371/journal.pone.0238699 (PMC7482915; doi:10.1371/journal.pone.0238699)
Supplement: S2 Appendix — (PDF) [file pone.0238699.s002.pdf]

Maslach Burnout Inventory (MBI)

question	Never	A few times in year	Once a month	Afew times per month	Evry week	Afe w times in wee k	Every day
**Emotional Exhaustion**	**0**	**1**	**2**	**3**	**4**	**5**	**6**
**1. I feel emotionally drained by my work**							
**2. I feel used up at the end of the workday**							
**3. I feel fatigued when I get up in the morning and have to face another day on the job**							
**4. Working with people all day long requires a great deal of effort**							
**5. I feel burned out from my work**							
**6. I feel frustrated by my job**							
**7. I feel I work too hard at my job**							
**8. It stresses me too much to work in direct contact with people**							
**9. I feel like I'm at the end of my rope**							
**10.I feel I treat some recipients as if they were impersonal 'objects**							
**II. Depersonalization**							
**11.I've become more callous toward people since I've taken this job**							
**12.I worry that this job is hardening me emotionally**							
**13.I do not really care what happens to some recipients**							
**14.I feel recipients blame me for some of their problems**							
**15.I can easily understand how my recipients feel about things**							
**III. Personal achievement**							
**16. I feel I'm positively influencing other people's lives through my work**							
**17.I feel very energetic**							
**18. I can easily create a relaxed atmosphere with my recipients**							

**19.I feel exhilarated after working closely with my recipients**							
**20. In my work, I deal with emotional problems very calmly**							
**21.I have accomplished many worthwhile things in this job**							
**22.I deal very effectively with the problems of my recipients**							

SCORING RESULTS – INTERPRETATION SCALE

Section A: Emotional Exhaustion

Emotional exhaustion measures feelings of being emotionally overextended and exhausted by one's work.

- ☐ Total 19 or less: Low-level burnout
- ☐ Total between 19 and 26 inclusive: Moderate burnout
- ☐ Total over 26: High-level burnout

Section B: Personal Accomplishment

Personal accomplishment measures feelings of competence and successful achievement in one's work.

- ☐ Total 34 or less: High-level burnout
- ☐ Total between 34 and 39 inclusive: Moderate burnout
- ☐ Total greater than 39: Low-level burnout

Section C: Depersonalization

Depersonalization measures an unfeeling and impersonal response toward recipients of one's service, care treatment, or instruction.

- ☐ Total 6 or less: Low-level burnout
- ☐ Total between 6 and 9 inclusive: Moderate burnout
- ☐ Total of 10 and greater: High-level burnout

A high score in Emotional Exhaustion and Depersonalization, and a low score in Personal Accomplishment may indicate burnout. Note: Different people react to stress and burnout differently. This self-test is not intended to be a scientific analysis or assessment. The information is not designed to diagnose or treat your stress or symptoms of burnout.

Effort—Reward Imbalance Model 22

Effort items (1-6):

1. I have constant time pressure due to heavy work load
2. I have many interruptions and disturbances while performing my job
3. I have a lot of responsibility in my job
4. I am often pressured to work overtime
5. My job is physically demanding
6. Over the past few years my job has become more and more demanding

Reward items: (7-17):

7. I receive the respect I deserve from my superiors
8. I receive the respect I deserve from my colleagues
9. I experience adequate support in difficult situations
10. I am treated unfairly at work
11. My job promotion prospects are poor
12. I have experienced or expect to experience an undesirable change in my work situation
13. My employment security is poor
14. My current occupational position adequately reflects my education and training
15. Considering all my efforts and achievements receive the respect and prestige I deserve at work

16. Considering all my efforts and achievements my job promotion prospects are adequate

17. Considering all my efforts and achievements my salary income is adequate

Over-commitment (18-23)

18. I get easily overwhelmed by time pressures work

19. As soon as I get up in the morning, I start thinking about work problems

20. When I get home, I can easily relax and switch off work

21. People close to me say I sacrifice too much for my job

22. Work rarely lets me go it is still on my mind when I go to bed

23. If I postpone something that I was supposed to do today I will have trouble sleeping at the night

uses four-point Likert scales: 1. Strongly disagree 2. Disagree 3. Agree 4. Strongly agree

- with SIX items measuring effort,
- TEN measuring reward
- and six measuring over-commitment [3].

To identify ERI, the effort–reward ratio is calculated,

as follows:

$$ER = K * E \backslash R$$

$$K = 10 \backslash 6$$

where E and R are the effort and reward scores, respectively,

and k is a correction factor (k = 10/6))

ERI is present when ER # 1,

with ER <1 indicating an imbalance in favor of rewards

and ER >1 indicating an imbalance in favor of effort

Appendix 3: Rosenberg's self-esteem Questionnaire (RSES)

STATEMENT		Strongly Agree	Agree	Disagree	Strongly Disagree
1.	I feel that I am a person of worth, at least on an equal plane with others.				
2.	I feel that I have a number of good qualities.				
3.	All in all, I am inclined to feel that I am a failure.				
4.	I am able to do things as well as most other people.				
5.	I feel I do not have much to be proud of.				

6.	I take a positive attitude toward myself.					
7.	On the whole, I am satisfied with myself.					
8.	I wish I could have more respect for myself.					
9.	I certainly feel useless at times.					
10.	At times I think I am no good at all.					

Your score on the Rosenberg self-esteem scale is: .

Scores are calculated as follows:

- For items 1, 2, 4, 6, and 7:

```
Strongly agree  = 3

Agree  = 2
```

Disagree = 1

Strongly disagree = 0

-

- For items 3, 5, 8, 9, and 10 (which are reversed in valence):

Strongly agree = 0

Agree = 1

Disagree = 2

Strongly disagree = 3

-

The scale ranges from 0-30. Scores between 15 and 25 are within normal range; scores below 15 suggest low self-esteem.

Practice Environment Scale-Nursing Work Index (PES-NWI)

Nurse Participation in Hospital Affairs

1. Staff nurses are involved in the internal governance of the hospital (e.g., practice and policy committees
2. Career development/clinical ladder opportunity
3. Opportunities for advancement
4. Administration that listens and responds to employee concerns
5. A chief nursing officer which is highly visible and accessible to staff
6. Career development/clinical ladder opportunity
7. Nursing administrators consult with staff on daily problems and procedures.
8. Staff nurses have the opportunity to serve on hospital and nursing committees
9. A chief nursing officer equal in power and authority to other top-level hospital executives.

Nursing Foundations for Quality of Care

1. Use of nursing diagnoses
2. An active quality assurance program
3. A preceptor program for newly hired RNs
4. Nursing care is based on a nursing, rather than a medical, model
5. Patient care assignments that foster continuity of care, i.e., the same nurse cares for the patient from one day to the next

6. A clear philosophy of nursing that pervades the patient care environment
7. Written, up-to-date nursing care plans for all patients.
8. High standards of nursing care are expected by the administration
9. Active staff development or continuing education programs for nurses
10. Working with nurses who are clinically competent

Nurse Manager Ability, Leadership, and Support of Nurses

1. A nurse manager who is a good manager and leader.
2. A nurse manager who backs up the nursing staff in decision-making, even if the conflict is with a physician
3. Supervisors use mistakes as learning opportunities, not criticism
4. A supervisory staff that is supportive of the nurses
5. Praise and recognition for a job well done

Staffing and Resource Adequacy

1. Enough staff to get the work done.
2. Enough registered nurses to provide quality patient care
3. Adequate support services allow me to spend time with my patients.
4. Enough time and opportunity to discuss patient care problems with other nurses

Collegial Nurse-Physician Relations

1. A lot of team work between nurses and physicians.

2. Physicians and nurses have good working relationships

3. Collaboration (joint practice) between nurses and physicians.

Likert:

1. . strongly disagree
2. Disagree
3. Agree
4. Strongly agree

This composite score calculated as the mean of the subscale scores provided a single continuous measure to compare practice environments. Lake et al (2002)(14) have developed a three level classification (favorable, mixed and unfavorable)

To assist in interpreting the composite subscale scores.

-Favorable settings were those where subscale scores were greater than 2.5.for four or five subscales.

-Mixed settings had two or three subscales with scores greater than 2.5 and

-Unfavorable settings none or one subscale.
